# Failure to reappraise: Malevolent creativity is linked to revenge ideation and impaired reappraisal inventiveness in the face of stressful, anger-eliciting events

**DOI:** 10.1080/10615806.2021.1918682

**Published:** 2021-04-26

**Authors:** Corinna M. Perchtold-Stefan, Andreas Fink, Christian Rominger, Ilona Papousek

**Affiliations:** Department of Psychology, University of Graz, Graz, AUT

**Keywords:** Cognitive reappraisal, malevolent creativity, revenge, coping, emotion regulation

## Abstract

**Background and objectives:** The complexities of daily life often necessitate creative ideas to successfully cope with negative social situations. This study investigated the relationship of two types of creativity that may be elicited by similar contexts but are associated with different goals and impact of ideas: reappraisal inventiveness (the capability to generate manifold reappraisals for negative situations) and malevolent creativity, capturing the inventiveness in intentionally harming others.

**Design and methods:** In 73 women, these variables were assessed by performance tests depicting real-life, anger-eliciting situations. Additionally, participants reported their trait anger and depressive symptoms.

**Results:** Inventiveness (ideational fluency) was positively correlated between the two tasks, probably indicating shared divergent thinking demands. A more intricate pattern emerged for quality aspects of generated ideas. Participants inventing particularly harmful ideas for damaging others generated fewer valid reappraisals and displayed less problem-oriented thinking during reappraisal. Greater inventiveness in damaging others was linked to more revenge-related ideation during reappraisal attempts, which also correlated with self-reported depressive symptoms.

**Conclusions:** A higher capacity for malevolent ideation may potentially hamper successful coping with stressful, anger-eliciting events and, as a result, may advance an adverse spiral of reinforcement. Considering these links may help tailor psychotherapeutic interventions to individuals’ specific predispositions.

## Introduction

In daily life, individuals are often required to come up with rather creative ideas for successfully coping with negative social situations. Interestingly, such situations, particularly anger-eliciting ones, may prompt two types of “affective creativity” that are very different in their potential impact: reappraisal inventiveness (the capability for generating manifold reappraisals in order to feel better about a situation) and malevolent creativity (inventiveness in harming others). In the present study, we investigated how individuals’ potential for malevolent creativity may relate to their capability and preferred strategies for generating suitable reappraisals for provocative contexts, following the idea that a high proficiency in harm-based problem-solving strategies may have maladaptive consequences in the context of emotion regulation.

### Inventiveness in cognitive reappraisal generation

Among various emotion regulation strategies, cognitive reappraisal entails the deliberate re-interpretation of an emotionally evocative event, and by adoption of different situational perspectives, changing its emotional impact (Ellsworth & Scherer, 2003; Gross & John, 2003; Lazarus & Folkman, 1984). The capacity to ad-hoc generate manifold alternative appraisals of stressful events is referred to as reappraisal inventiveness (Weber et al., 2014). Higher inventiveness is assumed to facilitate successful reappraisal implementation in everyday negative situations: The capability to readily generate a large and diverse pool of reappraisals of a given situation should translate into having a suitable reappraisal on hand when it is needed; and into being able to select from a broad repertoire of potential reappraisals the one that can be most effectively implemented in a given situation (Perchtold et al., 2019a; Wisco & Nolen-Hoeksema, 2010). This is particularly important in new or unexpected adverse situations, in which relying on one's habitual strategy for cognitive reappraisal is not sufficient (Fink et al., 2017; Papousek et al., 2017). This general idea is corroborated by empirical research showing that individuals generating a higher number and certain quality of reappraisal ideas for stressful, anger-eliciting events reported less chronic stress experience, and a lower proneness for hostile and suspicious thoughts (Perchtold et al., 2018b, 2019a).

In cognitive terms, reappraisal inventiveness resembles a competence that is commonly referred to as creative potential (the ability to produce multiple and alternative, preferably original yet practicable solutions to an open-ended problem; Runco & Acar, 2012; Runco & Jaeger, 2012), yet embedded in an affective context (Fink et al., 2017, 2018; Perchtold et al., 2018a). The similarity of neurocognitive processes involved in reappraisal inventiveness and creativity, most prominently the flexible generation of new perspectives and solutions and overriding the most obvious responses in favor of less apparent associations, was also substantiated empirically (Fink et al., 2017; Perchtold et al., 2018a; Rominger et al., 2018).

### Malevolent creativity

Another example of creative potential also prominent in the affective domain and possibly prompted by similar provocative contexts as cognitive reappraisal, is referred to as malevolent creativity. Malevolent creativity denotes creative thinking ability that is intentionally used to hurt, sabotage, or damage others (Cropley et al., 2008; 2010). It has been noted that individuals demonstrate a great amount of originality and ingenuity when it comes to destructive actions, both on the larger (terrorism, war strategies, criminal enterprise) and smaller scale (deception, harassment, bullying, or theft; e.g., Beaussart et al., 2013; Cropley, 2010; Harris et al., 2013). In terms of behavioral correlates, greater malevolent creativity was found to be positively related to maladaptive personality traits like antagonism and various aspects of aggressive behavior (Hao et al., 2016; Harris & Reiter-Palmon, 2015; Perchtold-Stefan et al., 2020).

Taken together, the inventiveness in finding suitable reappraisals of situations and the inventiveness in intentionally harming others undoubtedly share cognitive features linked to an individual's general potential for creativity. However, while in both cases relevant ideas may be instigated by unfair or provocative social situations that elicit anger, it is also obvious that the goals inherent in those ideas differ fundamentally. Finally, they may have a profoundly different impact on psychological functioning. This gives rise to the question how malevolent creativity and reappraisal inventiveness may relate to each other. To date, no research directly addressing this question exists. Some indirect indication was provided by Harris et al. (2013) who found higher malevolent creativity associated with lower emotional intelligence; and lower intra- and interpersonal emotional skills were reported for specific populations presumably higher in malevolent ideation (e.g., convicted criminals; Sharma et al., 2015). Additionally, individuals more readily generated malevolent ideas when in an angry mood or when facing social stress or provocative circumstances (Baas et al., 2019; Perchtold-Stefan et al., 2020).

#### The present study

We used two separate performance tests to assess reappraisal inventiveness and the inventiveness in intentionally harming others. In the Reappraisal Inventiveness Test (RIT; Weber et al., 2014) participants are asked to generate multiple reappraisals for stressful, anger-eliciting situations that may reduce their anger. The RIT scores overall inventiveness (ideational fluency, total number of ideas) as well as the propensity to produce certain types of appraisals (e.g., problem-oriented thinking or finding positive aspects). Likewise, the Malevolent Creativity Task (MCT; Perchtold-Stefan et al., 2020) scores inventiveness in terms of the number of ideas as well as quality indicators: the degree of malevolence (harmfulness) and originality (uniqueness) of generated ideas.

We followed two main research questions. First, we were interested in the relationship between malevolent creativity and the overall inventiveness in reappraising anger-eliciting situations (in terms of ideational fluency; RQ1). Here, we considered different outcomes for the fluency and the quality of ideas in the malevolent creativity test. Given that both abilities draw on creativity-related cognitive processes, the fluency in generating ideas in both tests was expected to be positively correlated. Greater ability to produce malevolent ideas that are more harmful and more original, on the other hand, may be related to a stronger habitual bias toward negativity and inflicting damage. Thus, it may show a negative correlation with reappraisal inventiveness, which by definition implicates the fluent generation of ideas involving a change of perspective away from resentment.

Second, we investigated the relationship between malevolent creativity and individuals’ propensity to produce a certain type of ideas during volitional reappraisal of anger-eliciting events (RQ2). In its elaborate scoring scheme, the RIT differentiates between different types of appraisals that are divided into four main categories: problem-oriented thinking, positive re-interpretation, de-emphasizing (detached reappraisal), and revenge-related ideation (Weber et al., 2014). While the latter is not usually regarded a valid reappraisal, studies with the RIT (Weber et al., 2014; also see Perchtold et al., 2018b) have shown that individuals regularly incorporate vengeful thoughts into their reappraisal attempts nevertheless; and according to the standard coding they are included in the total number of ideas constituting the fluency indicator of reappraisal inventiveness (Weber et al., 2014).

Important to note, in the RIT, revenge-related ideation is never explicitly instructed or encouraged and goes against the prevalent goal of finding less negative contextual interpretations. However, with frequent repetition in the same context, response strategies may eventually become a habitual response to relevant situations (Hertel, 2004). As a result, the greater availability of malevolent ideas in individuals with high expressions of malevolent creativity may impact their spontaneous way of dealing with stressful situations in everyday life; and they may also follow this track of ideation when they are actually dedicated to find a helpful reappraisal for an anger-eliciting event. In other words, based on their proficiency in malignant ideation, malevolently creative individuals may be less able to generate suitable, less negative re-interpretations, particularly in contexts where they experience anger due to harmful or negligent actions of a wrongdoer. Thus, if a positive relationship between malevolent creativity and revenge-ideation during volitional reappraisal is established, it may signal generalization of harm-based problem-solving strategies to the context of emotion regulation, where it may not be adaptive. For the present study, we hypothesized that due to greater availability of malevolent cognition, individuals with higher inventiveness in intentionally harming others may also show a stronger spontaneous propensity for revenge-related thoughts when trying to find anger-reducing reappraisals.

Based on recent findings that some strategies for cognitive reappraisal may be more adaptive than others as regards implications for affective functioning (e.g., Perchtold et al., 2018b; Shiota & Levenson, 2012), supplementary analyses included correlations between the use of different types of reappraisal strategies in the RIT and self-reported depressive experiences and trait anger.

## Methods

### Participants

The required sample size was estimated a priori with G*Power (α = .05, 1−β = 0.80). Effect sizes observed in previous relevant research suggested a minimum of 65 participants for a multiple regression approach (based on correlations of .37 to up to .62 between general creative potential and reappraisal inventiveness or malevolent creativity; Fink et al., 2017; Perchtold-Stefan et al., 2020; Rominger et al., 2018). The final sample comprised 73 participants, aged between 18 and 37 years (*M* = 23.9, *SD* = 4.3). All participants were university students enrolled in various fields and female. For this first investigation into cognitive reappraisal and malevolent creativity, we decided on a homogenous, all-female sample in order to reduce variance due to prevalent gender differences in the context of emotion regulation (see Nolen-Hoeksema, 2012) and anger reappraisal specifically (also see Fink et al., 2017), which is meant to ensure comparability with previous studies linking the RIT to aspects of well-being and cognition (see Fink et al., 2017; Papousek et al., 2017; Perchtold et al., 2018b, 2019b). Participants were recruited online via social media, and offline via posters at several university campuses. Interested individuals were phoned to check for exclusion criteria (drug use, psychoactive medication, neurological/psychiatric history, previous experience with the behavioral tests) and to arrange an appointment. In order to avoid transfer effects between behavioral tests, testing took place in two different sessions within a time span of at minimum, one week and at maximum, two weeks. Out of 85 initially contacted individuals, six failed to show up at their scheduled appointments and five more dropped out after the first session. One participant was excluded from data analysis after testing due to non-compliance with test instructions. The study was approved by the local authorized ethics committee. Participants gave their written consent to participate in the study. The Malevolent Creativity Test was completed at session one. At session two, participants completed the emotional trait questionnaires before working on the Reappraisal Inventiveness Test.

### Malevolent creativity

The Malevolent Creativity Test (MCT; Perchtold-Stefan et al., 2020) consisted of four realistic, open-ended problems depicting different sorts of unfair and likely anger-eliciting behavior from peers/associates, as it was shown that malevolent creativity is primarily featured in unfair and provocative contexts (e.g., Baas et al., 2019; Harris & Reiter-Palmon, 2015). In the money item of the MCT, for example, participants face the following scenario:

Your neighbor asks you to help them with renovations in their flat and offers to pay you for your troubles. Since you are currently low on money, you agree. After the work is done, you ask them for the payment they promised. However, your neighbor insists that such an agreement never took place and you just imagined the whole thing.

Participants were instructed to generate as many original ideas as possible to react to the unfair behavior depicted in these situations in order to get back at or sabotage the wrongdoer. These instructions conform to the definition of malevolent creative cognition as the generation of novel ideas with the goal to deliberately harm and damage others (e.g., Cropley et al., 2008). A practice item was given prior to the task. Each situation was presented on a computer screen for 30 s and was supplemented by a matching photograph. Participants were told to imagine the situation happening to them and to try and picture it as vividly as possible. Subsequently, at the appearance of a white question mark on screen, participants then generated as many original ways as possible to sabotage that person/take revenge for the unfair treatment on a sheet in front of them. After the allotted time of 3 min, a short tone indicated a new vignette appearing on the screen. At the end, participants rated how angry individuals would feel when confronted with the depicted situations in real life (7-point scales ranging from 0 “not angry at all” to 6 “very angry”; *M* = 4.33, *SD* = 0.87, α = .83). In one-sample t-tests, anger ratings of all four vignettes differed significantly from zero (t-values from 20.32–31.21, all *p*-values <.001), indicating that all depicted vignettes constituted situations in daily life that may evoke malevolent creativity (e.g., Harris & Reiter-Palmon, 2015; James et al., 1999).

In line with previous studies on negative and malevolent creativity (Harris & Reiter-Palmon, 2015; Kapoor & Khan, 2017; Perchtold-Stefan et al., 2020), we used scores for ideational fluency (number of malevolent ideas), malevolence, and originality of ideas generated in the MCT. On average, participants generated a total of *M* = 17.41 ideas (*SD* = 5.48). However, only ideas that met the instructions of being (at least slightly) malevolent as evaluated by four independent raters were scored as valid (ICC = .99; *M* = 91.7% of generated ideas, *SD* = 12.2%), resulting in the ideational fluency score, denoting the inventiveness in intentionally harming others. Malevolence was scored on a 4-point Likert scale by the same four raters, with 1 indicating a slightly harmful idea (e.g., talking badly with friends about the wrongdoer) and 4 indicating a very harmful idea (e.g., hiring some people to kidnap the wrongdoer and beat some sense into them; ICC = .89). Originality (uniqueness) of generated ideas was rated on a 4-point Likert scale by the same four raters (1 = not original, 4 = very original; cf. Consensual Assessment Technique; Amabile, 1987). Inter-rater reliability was ICC = .89. See Table 1 for descriptive statistics of the MCT.

**Table 1.** Descriptive statistics of scores in the two performance tests.

	M	SD	Min	Max
Reappraisal inventiveness (RIT)				
Total n of ideas (fluency)	35.88	14.21	5	64
Problem-oriented thinking	11.82	7.59	0	35
Positive re-interpretations	6.40	5.30	0	23
De-emphasizing	13.68	11.73	0	48
Revenge-related ideation	3.49	3.91	0	19
Malevolent creativity (MCT)				
Total n of ideas (fluency)	16.17	5.61	1	33
Malevolence	2.11	0.35	1	2.66
Originality	2.03	0.42	1	3.09

Note*.* M = mean value; SD = standard deviation; Min = minimum, Max = Maximum

### Reappraisal inventiveness

The Reappraisal Inventiveness Test (RIT; Weber et al., 2014) confronts individuals with stressful, everyday situations they can easily imagine happening to them and that require the generation of alternative appraisals in order to downregulate the experienced negative emotion, in this case, anger. In line with cognitive emotion theories, these situations depict harmful behavior of another person, while at the same time, they are ambiguous on whether this behavior occurs willingly or carelessly. In the plant item of the RIT, for instance, participants face the following situation:

You arrive at our apartment after having been on a long vacation. You had asked a friend of yours to water the plants while you were gone. Now you see that most of your plants have died. You call your friend. They tell you that the distance to your apartment was too long for them to water your plants as agreed (see Weber et al., 2014, p. 360).

Items are supplemented by matching photographs in order to make the depicted situations more vivid. The present study used six RIT items, all of which were previously demonstrated to be sufficiently anger evoking (see Fink et al., 2017; Papousek et al., 2017; Perchtold et al., 2019b). Each vignette was presented on a computer screen for 20 s. Participants were instructed to imagine the situation happening to them and at the appearance of a white question mark on screen, to generate as many different ways as possible to think about or appraise the situation in a way that diminishes anger. After the allotted time of 3 min, a short tone indicated a new vignette appearing on the screen.

The RIT quantifies reappraisal inventiveness as the total number of generated nonidentical reappraisal ideas when attempting to downregulate anger by means of reappraisal (Weber et al., 2014). It was independently rated by two experienced researchers, with a resulting intraclass correlation (ICC) of .99 (see previous satisfying interrater reliabilities of ICC = .90 to .99 in Fink et al., 2017; Papousek et al., 2017; Perchtold et al., 2019a; Weber et al., 2014). In order to determine individuals’ propensity for specific types of reappraisal ideas, they were categorized according to the standard category scheme of the RIT (Weber et al., 2014). The four main strategies coded by the RIT are problem orientation (referring to action planning and finding ways to reduce harm; ICC = .97), positive reinterpretation (perspective change in terms of generating positive aspects and casting disadvantages as advantages; ICC = .96), de-emphasizing (distancing oneself and trivializing the impact of the situation; ICC = .98), and revenge-related ideation (finding ways to get even with the wrongdoer; ICC = .94). Ideas not matching these four categories were coded as “other” and excluded from analyses due to lack of answers generated by the participants. See Table 1 for descriptive statistics of the RIT.

### Self-report measures of affective functioning

*Depressive daily life experiences* were assessed with the Center for Epidemiologic Studies Depression Scale (CES-D, German version; Hautzinger & Bailer, 1993). The CES-D comprises 20 items, rated from 0 (rarely or none of the time – less than 1 d) to 3 (most or all the time – 5–7 days; α = .82). Scores ranged from 0 to 22 (*M* = 9.36, *SD* = 4.73).

*Individuals’ propensity to experience anger* was measured with the German version of the Spielberger State-Trait Anger Expression Inventory (STAXI, Schwenkmezger et al., 1992, trait anger subscale, 10 items). Items are rated on a four-point Likert scale, from 1 (never or rarely) to 4 (most or all the time; α = .73). Scores ranged from 10 to 26 (*M* = 17.15, *SD* = 3.58):

### Statistical analysis

In order to determine whether malevolent creativity was linked to reappraisal inventiveness in terms of the fluency of ideas for reappraising anger-eliciting situations, we computed Pearson correlations between the three indices of the MCT (ideational fluency, malevolence, and originality) and the number of ideas (fluency) in the RIT (RQ 1).

Next, to answer RQ2 whether malevolent creativity was linked to a greater proneness for generating specific types of ideas during volitional reappraisal of anger-eliciting events, we used three standard multiple regression analyses: The numbers of ideas classed as each of the four main strategies applied in the RIT (problem-oriented thinking, positive re-interpretation, de-emphasizing, revenge ideation) were simultaneously entered as predictors. The semipartial correlations gained in these multiple regression analyses allowed to determine whether malevolent creativity was related to the predominant implementation of certain strategies during reappraisal compared to others, and independently from the individual's overall inventiveness. Fluency, originality, and malevolence scores of the MCT were respectively entered as dependent variables.

As supplementary analyses, the same multiple regression approach from RQ2 was used to investigate whether the relative preferred use of any of the four RIT strategies was linked to depressive experiences and trait anger, respectively. The statistical assumptions for the multiple regression models (i.e., ratio of cases to independent variables, normality, independence of errors, homoscedasticity, linearity, and absence of multicollinearity) were met. Since greater malevolent creativity may shape habitual reappraisal responses over time, we additionally performed moderation analysis for significant effects observed in the regression models, to see whether the age of participants moderated associations between malevolent creativity and reappraisal inventiveness. For this purpose, the SPSS macro PROCESS set to Model 1 (Hayes & Preacher, 2014) was used. Results were considered statistically significant, if *p* < .05 (two-tailed). Exact *p*-values are given for all analyses to make them fully open to scrutiny.

## Results

Descriptive statistics for the RIT and the MCT are reported in Table 1.

### Relationship between malevolent creativity and ideational fluency while finding reappraisals of anger-eliciting situations

Higher ideational fluency in the Malevolent Creativity Test (MCT) was correlated with higher fluency of findings ideas for reappraising anger-eliciting situations (*r* = .33, *p* = .005). However, production of malevolent ideas that were rated as more harmful (MCT) was correlated with lower reappraisal inventiveness (*r* = -.24, *p* = .038). The correlation between originality of ideas generated in the MCT and reappraisal inventiveness was non-significant (*r* = -.11, *p* = .377). Individuals demonstrating a higher number of ideas in the MCT also scored higher on malevolence of ideas (*r* = .27, *p* = .038; originality: *r *= .21, *p* = .076). Malevolence and originality were correlated at *r* = .59 (*p* < .001).

### Relationship between malevolent creativity and strategies applied during volitional reappraisal of anger-eliciting events

Higher ideational fluency in the MCT was independently associated with a greater share of revenge-related ideas when trying to find anger-reducing reappraisals (*sr* = .26, *p* = .023; see Table 2). Greater malevolence of ideas in the MCT was independently associated with a lower share of problem-oriented thinking during volitional reappraisal of anger-eliciting situations (*sr* = -.27, *p* = .024; see Table 3). Associations between the use of the four strategies applied in the RIT and originality of malevolent ideas in the MCT mirrored the above reported pattern (revenge-related thoughts: *sr* = .23, *p* = .040; problem-oriented thinking: *r* = -.32, *p* = .006, see Table 4). No unique contributions of the share of positive re-interpretations or de-emphasizing reappraisals to the MCT scores were observed (all *p*’s >.088, see Tables 2–4).

**Table 2.** Fluency in Malevolent Creativity (MCT) and Use of Specific Strategies During Reappraisal of Anger-eliciting Events (RIT).

Reappraisal strategies	R²	r	*p*	sr	B	SE	β	*p*	95% CI [LL, UL]
Problem-oriented thinking	.14	.08	.511	.06	.05	.09	.07	.626	[-.14, .23]
Positive re-interpretations		.20	.092	.19	.21	.13	.21	.101	[-.04, .47]
De-emphasizing		.16	.179	.14	.08	.06	.17	.222	[-.05, .20]
Revenge-related ideation		.21	.070	.26	.39	.17	.29	.023	[.06, .73]

Note*.* Standard multiple regression analysis; *F*(4,68) = 2.67, *p* = .038; *R*² = proportions of variance explained by the model in total, *r* = Pearson correlation; *sr* = semipartial correlation, *B* = unstandardized beta weight, *SE* = standard error for *B, β* = standardized beta weight, *CI* = confidence interval, *LL* = lower limit, *UL* = upper limit.

**Table 3.** Harmfulness of Malevolent Ideas (MCT) and Use of Specific Strategies During Reappraisal of Anger-eliciting Events (RIT).

Reappraisal strategies	R²	r	*p*	sr	B	SE	β	*p*	95% CI [LL, UL]
Problem-oriented thinking	.09	-.20	.092	-.27	-.02	.01	-.31	.024	[-.03, -.01]
Positive re-interpretations		-.13	.263	-.06	-.01	.01	-.07	.603	[-.02, .01]
De-emphasizing		-.10	.393	-.15	-.01	<.01	-.19	.187	[-.01, .00]
Revenge-related ideation		.02	.889	.06	-.01	.01	.07	.602	[-.02, .03]

Note*.* Standard multiple regression analysis; *F*(4,68) = 1.72, *p* = .157; *R*² = proportions of variance explained by the model in total, *r* = Pearson correlation; *sr* = semipartial correlation, *B* = unstandardized beta weight, *SE* = standard error for *B*, *β* = standardized beta weight, *CI* = confidence interval, *LL* = lower limit, *UL* = upper limit.

**Table 4.** Originality of Malevolent Ideas (MCT) and Use of Specific Strategies During Reappraisal of Anger-eliciting Events (RIT).

Reappraisal strategies	R²	r	*p*	sr	B	SE	β	*p*	95% CI [LL, UL]
Problem-oriented thinking	.13	-.20	.098	-.32	-.02	.01	-.38	.006	[-.04, -.01]
Positive re-interpretations		.05	.663	.16	.02	.01	.18	.160	[-.01, .04]
De-emphasizing		-.07	.557	-.20	-.01	.01	-.24	.088	[-.02, .00]
Revenge-related ideation		.13	.285	.23	.03	.01	.26	.040	[.01, .06]

Note*.* Standard multiple regression analysis; *F*(4,68) = 2.57, *p* = .046; *R*² = proportions of variance explained by the model in total, *r* = Pearson correlation; *sr* = semipartial correlation, *B* = unstandardized beta weight, *SE* = standard error for *B*, *β* = standardized beta weight, *CI* = confidence interval, *LL* = lower limit, *UL* = upper limit.

### Supplementary analyses

A greater share of revenge-related ideas when trying to find anger-reducing reappraisals (RIT) was independently associated with a greater amount of depressive experiences (*sr* = .28, *p* = .018; zero order correlation *r* = .25, *p* = .035*; F*(4,68) = 1.67, *p* = .168). No unique contributions of other strategies during volitional reappraisal to depressive experiences were observed (problem-oriented thinking: *sr* = -.13, *p* = .275; positive re-interpretation: *sr* = .13, *p* = .277; de-emphasizing: *sr* = -.10, *p* = .412). Trait anger did not show significant associations with use of reappraisal strategies (*F*(4,68) = 0.818, *p* = .518, see Supplementary File for details). Malevolent creativity did not directly correlate with the measures of affective functioning (depressive experiences, fluency: *r* = .05, *p* = .679; malevolence: *r* = .18, *p* = .133; originality: *r* = .08, *p* = .511; trait anger, fluency: *r* = -.02, *p* = .901; malevolence: *r* = .12, *p* = .315; originality: *r* = -.14, *p* = .254). No moderation effects of age on the link between malevolent creativity and reappraisal inventiveness or types of reappraisals were observed (*p*’s for all interaction effects >.10).

## Discussion

The present study investigated the assumption that individuals’ inventiveness in intentionally hurting or damaging others may be linked to the easiness with which they find alternative reappraisals for stressful, anger-eliciting events as well as to the types of appraisals they tend to use in this regard.

First, participants generating a higher number of ideas in the malevolent creativity task also displayed greater fluency of ideas when trying to find reappraisals of anger-eliciting situations. The positive correlation corroborates the notion that both abilities draw on similar, creativity-related cognitive processes that allow for fluent generation of ideas in the context of social problem solving. It may also signal a general proneness for divergent thinking irrespective of task or problem (see Harris & Reiter-Palmon, 2015), which is supported by moderate to large correlations between (non-affective) verbal creativity and malevolent creativity (Perchtold-Stefan et al., 2020) as well as between verbal creativity and reappraisal inventiveness (Fink et al., 2017; Weber et al., 2014). Another obvious overarching competency may be (i.e., non-affective, not necessarily creative) verbal fluency.

However, the other observed pattern in this study suggests that malevolent creativity may be related to the capacity for finding alternative reappraisals via qualitative aspects. A higher degree of malevolence (harmfulness) of ideas generated with the purpose of doing harm to another person was correlated with less fluent generation of ideas when it came to volitional reappraisal of anger-eliciting situations. This indicates that a higher capacity to produce particularly malevolent solutions to social problems may hamper the ability to invent other flexible situational re-interpretations that help cast things in less negative ways. Interestingly, De Dreu and Nijstad (2008) reported that individuals prone to conflict-related cognition such as hatred or hostility, while displaying higher creativity in competitive, antisocial settings, showed lower ideational fluency and diversity in cooperative, pro-social contexts. Applied to cognitive reappraisal, this would suggest that a higher capacity for harmful ideation may potentially inhibit additional prosocial perspectives on anger-eliciting scenarios, like finding alternative explanations for the wrongdoers’ behavior, altruistic thoughts, or appreciation of the wrongdoer (Weber et al., 2014). Fitting this interpretation, lower reappraisal inventiveness for anger-evoking events was previously found in individuals with a more hostile and suspicious interpretation bias (Perchtold et al., 2019b). On a general note, reappraisal inventiveness denotes individuals’ capacity to ad hoc generate many different cognitive re-interpretations of adverse events (Weber et al., 2014). While in daily life, it may initially seem more relevant to produce one high-quality reappraisal than a variety of reappraisals to effectively reduce the impact of aversive events, it can be argued that having a broad repertoire of potential reappraisals readily available increases the likelihood to select the most effective one for a specific context (e.g., Perchtold et al., 2019a; Wisco & Nolen-Hoeksema, 2010). Accordingly, it may be suggested that a more exclusive focus on malevolent solutions for provocative situations may restrict individuals’ pool of eligible reappraisal ideas, which in turn, reduces reappraisal success, particularly in novel situations that exceed routines (Papousek et al., 2017).

The present study further allowed a deeper insight into the structure of individuals’ coping attempts when faced with annoying, potentially stressful social situations: The applied test for reappraisal inventiveness provided indicators for individuals’ preference for specific types of reappraisals over others, which may indicate a more automatic propensity or proneness to activate certain contents and schemata during reappraisal generation. Supporting our hypothesis, higher malevolent creativity was independently associated with a higher propensity for revenge-related ideation during intentional reappraisal of negative social situations. This relationship indicates that individuals highly capable of producing malevolent ideas for the purpose of revenge when explicitly asked to do so may also spontaneously employ their malevolent ideation as a strategy when trying to use reappraisal for coping with provocative events. Previous research indicates that high malevolent creativity is at least partly grounded in hostile and antagonistic perceptions of reality (e.g., Hao et al., 2016; Harris & Reiter-Palmon, 2015; Lee & Dow, 2011; Perchtold-Stefan et al., 2020). According to our results, this antagonistic thinking may also drive malignant and vengeful problem-solving tactics in the context of anger reappraisal (e.g., for the RIT plant item: “*An eye for an eye”*, “*Stuffing the dead plant into the friend’s mailbox”*, “*Telling mutual friends what a horrible person they are”*). While speculative at this point, it is possible that individuals high in malevolent creativity may exhibit certain difficulty in inhibiting their malevolent problem-solving skills and aptitude for punishment in favor of alternative situational perspectives.

Additionally, greater malevolence and originality of ideas generated for hurting or damaging another person were independently associated with a lower propensity for problem-oriented thinking when attempting to cope with annoying social situations via reappraisal. It appears that malevolent creativity interferes not only with more versatile views of irritating events, but also with more emotionally neutral and rational solutions, that is, reappraisal ideas focused on possible actions and remedies for the incurred damage (e.g., for the RIT plant item: “*Planning to buy new plants or try and save the remaining ones”*). While we did not initially expect this relationship, it matches previous findings that individuals high in socially aversive personality traits (e.g., psychopathy, machiavellianism) that have also been linked to harm-based creativity (e.g., Jonason et al., 2017; Kapoor, 2015), reported lower preference for active problem-solving strategies in dealing with emotional distress (Birkás et al., 2016).

Greater preference for revenge-related ideation and a lower preference for problem-oriented thinking may have practical consequences when it comes to successful coping with difficult interpersonal situations. While this is the first study to directly scrutinize thoughts of revenge during cognitive reappraisal, broader literature suggests that fantasies as well as acts of revenge are linked to better mood repair after interpersonal rejection (Chester & DeWall, 2017) as well as improved self-efficacy and reduced levels of frustration (Haen & Weber, 2009; also see Seebauer et al., 2014). These short-term positive effects are likely the reason why individuals frequently exercise revenge fantasies during therapy (Seebauer et al., 2014) and why revenge is regularly employed for anger-relief during reappraisal attempts of other’s damaging actions (see Weber et al., 2014). Conversely, intervention studies classified revenge as an ineffective coping strategy, for instance, in light of findings that applying revenge ideation to cope with past bullying incidences did not decrease associated negative emotions, but decreased positive self-evaluation of victims (Copeland-Linder et al., 2012; Watson et al., 2016; but see Seebauer et al., 2014 for a different perspective). Moreover, it was strongly suggested that exacting revenge against a wrongdoer, against participants’ beliefs to the opposite, elevates feelings of anger instead of reducing them (Carlsmith et al., 2008). Similarly, self-reported anger with anger-inducing memories was highest after angry rumination that included retaliation as compared to normal reappraisal (Fabiansson et al., 2012). The prominent ruminative features of vengeful thoughts and their rehearsal of negative thought patterns are also discussed as a primary mechanism that links revenge ideation to an increased likelihood for negative affect and depression (e.g., Newman, 2011; Ysseldyk et al., 2019; also see Elshout et al., 2015). In this respect, Barcaccia et al. (2020) showed that negative affect directly mediated the link between high motivation for revenge and increased depressive symptoms. In the present study, greater use of revenge-related ideation was uncorrelated with trait anger, but positively correlated with self-reported depressive experiences. This generally corresponds to abovementioned accounts that using revenge ideation for affect regulation may be maladaptive over time, although the cross-sectional correlations in the present study certainly do not allow for an interpretation of causality. Accordingly, longitudinal investigations with larger samples are needed to determine the specific mental health effects of incorporating a greater share of revenge ideation when trying to cope with anger evoking events.

In any case, however, the present findings indicate that high inventiveness in ways to do harm to others can be an obstacle to productive reappraisal ideas for irritating social circumstances. From this, important implications may be deduced for psychotherapeutical settings aiming at encouraging patients to use and practice finding alternative reappraisals in relevant interpersonal situations. In conflict-prone patients or patients with a relevant criminal history who are likely high in malevolent creativity, it may be helpful to first direct the attention to the patient's routine cognition related to their particular ability and help them to purposefully inhibit the prepotent revenge ideation, in order to clear the way for using their inventiveness for the generation of constructive ideas for conflict resolution. Additionally, understanding that malevolent ideation may be more easily accessible to some individuals may help to adequately manage revenge fantasies that quite regularly occur in psychotherapy (Seebauer et al., 2014).

### Limitations and future directions

Several limitations of the present study should be acknowledged. First, due to the correlational/cross-sectional design of the present study, the direction of effects cannot be directly inferred. While it seems intuitive to argue that individuals well versed in finding creative ways to inflict harm on others would also automatically use their harm-based cognitions for regulating anger in the face of provocation, circular mechanisms are possible and should be investigated. Secondly, results are based on an all-female student sample, which naturally limits the generalizability of findings. For a replication in mixed-sex samples, we would assume that the link between malevolent creativity and use of revenge-related ideation during reappraisal is even stronger in men, given that men often display higher malevolent creativity than women (e.g., Harris & Reiter-Palmon, 2015; Lee & Dow, 2011; Perchtold-Stefan et al., 2020) and report a higher level of revenge fantasies with regard to past injustice (Goldner et al., 2019). Additionally, although study sessions were separated by a minimum of seven days, our experimental design always put the MCT before the RIT, which may raise potential concerns of order effects. However, we are confident that malevolent creativity solicited at session one did not inflate revenge ideation in the RIT at session 2, since the number of revenge-related ideas in the present study (∼10%) is highly similar to previous RIT studies without malevolent tasks (see Perchtold et al., 2018b, 2019b). Still, future studies are needed to replicate our results in randomized designs that fully control for potential order effects, in addition to using a more diverse set of tasks. We did not observe significant moderating effects of age, which would have strengthened our idea that higher malevolent creativity may stimulate maladaptive, revenge-related reappraisal attempts over time. However, the age range in our sample of young university students was rather limited. This highlights the need for a replication and extension of our proposed mechanisms in samples of different ages. Another interesting avenue for future research is the fact that despite specific instructions to the contrary, ∼8% of answers in the MCT were non-malevolent, and even partly comprised problem-oriented reappraisals for dealing with the provocative situations. While in the present study, the number of respective answers was too low for meaningful analyses (∼3%), it will be interesting to look at well-being and personality traits of individuals who unsolicitedly use reappraisal in situations that clearly prompt malevolent ideation. Lastly, both malevolent creativity and reappraisal inventiveness were assessed with laboratory-bound, performance-based instruments, which may raise questions on their ecological validity. However, Perchtold-Stefan et al. (2020) previously demonstrated that individuals with higher performance on the MCT also reported to engage in more malevolent creativity behaviors in daily life (e.g., lying, hurting others, playing tricks on others, Hao et al., 2016). Similarly, individuals’ spontaneous cognitive reappraisal generation in the RIT was closely related to health outcomes that suggest successful reappraisal implementation in daily life (Perchtold et al., 2018b, 2019a).

## Conclusion

Taken together, the present study demonstrated that individuals’ capacity for malevolent ideation is related to their implementation of cognitive reappraisals for stressful, anger-eliciting events. The overall pattern of results suggests that malevolent creativity may impede individuals’ coping with anger-eliciting events through impaired reappraisal skills in terms of attenuated ideational fluency and lesser use of problem-oriented thinking, probably contingent on intrusive revenge-related cognition. Failed coping and conflict resolution then may further stimulate malevolent ideation and thus advance an adverse spiral of reinforcement. In therapeutic practice, understanding these links may help to break the vicious circle and may help tailor interventions to an individual’s specific predispositions and needs.

## Data Availability

The data that support the findings of this study are available from the corresponding author, CPS, upon reasonable request.
